# Successful Deployment of High Flow Nasal Cannula in a Peruvian Pediatric Intensive Care Unit Using Implementation Science—Lessons Learned

**DOI:** 10.3389/fped.2018.00085

**Published:** 2018-04-11

**Authors:** Katie R. Nielsen, Rosario Becerra, Gabriela Mallma, José Tantaleán da Fieno

**Affiliations:** ^1^Department of Pediatrics, Critical Care Medicine, University of Washington, Seattle, WA, United States; ^2^Department of Global Health, University of Washington, Seattle, WA, United States; ^3^Departamento de Cuidados Intensivos Pediátricos, Instituto Nacional de Salud del Niño, Lima, Peru; ^4^Universidad Nacional Federico Villarreal, Lima, Peru

**Keywords:** high flow nasal cannula, implementation science, resource-limited setting, peru, pediatrics, acute respiratory failure

## Abstract

Acute lower respiratory infections are the leading cause of death outside the neonatal period for children less than 5 years of age. Widespread availability of invasive and non-invasive mechanical ventilation in resource-rich settings has reduced mortality rates; however, these technologies are not always available in many low- and middle-income countries due to the high cost and trained personnel required to implement and sustain their use. High flow nasal cannula (HFNC) is a form of non-invasive respiratory support with growing evidence for use in pediatric respiratory failure. Its simple interface makes utilization in resource-limited settings appealing, although widespread implementation in these settings lags behind resource-rich settings. Implementation science is an emerging field dedicated to closing the know-do gap by incorporating evidence-based interventions into routine care, and its principles have guided the scaling up of many global health interventions. In 2016, we introduced HFNC use for respiratory failure in a pediatric intensive care unit in Lima, Peru using implementation science methodology. Here, we review our experience in the context of the principles of implementation science to serve as a guide for others considering HFNC implementation in resource-limited settings.

## Introduction

Acute lower respiratory infections remain the leading cause of death outside the neonatal period for children less than 5 years of age, and the majority of these deaths occur in low- and middle-income countries (1). These large discrepancies in mortality are due, at least in part, to a lack of availability of basic supplies like oxygen and antibiotics and more advanced technology such as invasive and non-invasive mechanical ventilation (2–4). Despite efficacy of many types of advanced pediatric respiratory support (5, 6), widespread implementation of these technologies in resource-limited settings has lagged behind (7). Implementation science is a rapidly growing field dedicated to improving quality of health care delivery by incorporating evidence-based practices into routine care. Implementation science principles have guided the scale-up of interventions such as prevention of maternal-to-child transmission of HIV in resource-limited settings and could be applied to respiratory technologies to help ensure their success (8, 9).

High flow nasal cannula (HFNC) is an alternative form of non-invasive respiratory support with increasing use for respiratory failure in neonates, children, and adults (6, 10). Due to the non-occlusive nature of the nasal cannula, HFNC is easier to manage by bedside providers than CPAP or BiPAP because there is no need to readjust the interface to maintain a seal. In fact, HFNC has been used for pediatric patients outside the ICU setting with great success (11–13). In resource-limited settings where high patient:provider ratios limit clinicians’ ability to be at the bedside, the simple HFNC interface has the potential to be successful in supporting children with respiratory failure. This, as well as evidence that HFNC may be more comfortable for pediatric patients (14, 15), led us to pursue implementation of HFNC in the Pediatric Intensive Care Unit (PICU) at Instituto Nacional de Salud del Niño (INSN) in Lima, Peru. A prior study in Ghana demonstrated substantial challenges in sustaining CPAP in that resource-limited setting (16), so we decided to use implementation science principles to guide our deployment strategy. In this article, we describe our implementation science approach to guide others planning to introduce pediatric advanced respiratory care in resource-limited settings, so that they may avoid common pitfalls.

## Setting

**Figure 1:**
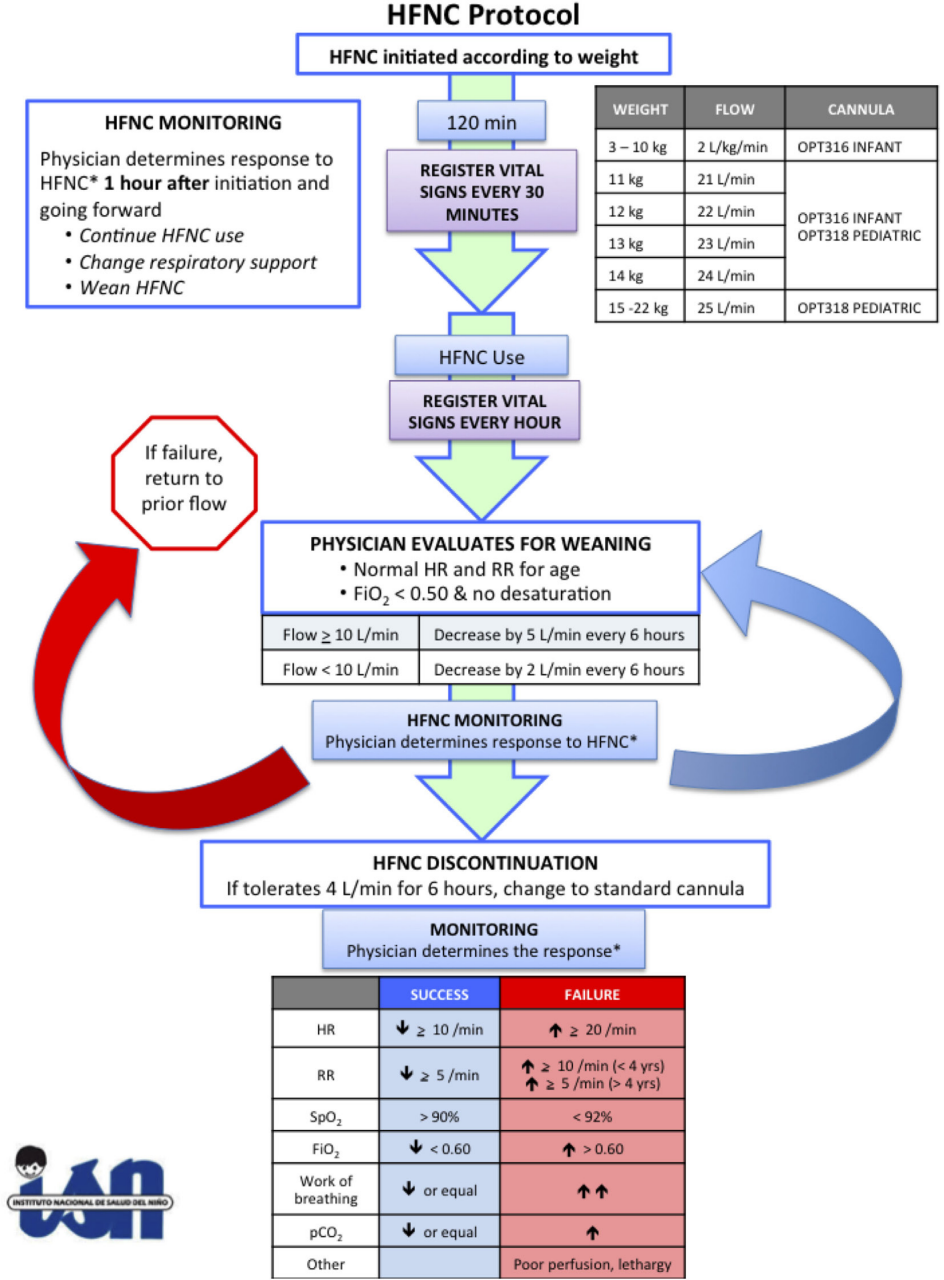
High flow nasal cannula protocol at Instituto Nacional de Salud del Niño.

Instituto Nacional de Salud del Niño is the largest freestanding children’s hospital in Peru with approximately 400 inpatient beds and 15 PICU beds. It is a major tertiary care referral center for children covered by the government insurance program, Seguro Integral de Salud. The PICU has approximately 400 admissions annually, of which, 50% are surgical and 50% medical, and a mortality rate of 18%. Children up to 18 years of age with any medical or surgical pathology may be admitted, although oncology and burn patients are typically admitted to other facilities, and postoperative cardiac surgery patients are admitted to the cardiac intensive care unit. Pediatric critical care physicians are in-house 24 h a day, 7 days a week. The nurse:patient ratio is 1:2, and there is one respiratory therapist available during day shift. HFNC was implemented as part of a larger research study to determine whether post-extubation use of HFNC would decrease the duration of invasive mechanical ventilation. According to the research protocol, all children less than 5 years of age who required invasive mechanical ventilation during the first 24 h of PICU admission were eligible for the study unless they had craniofacial malformations that would preclude HFNC use. Informed consent was obtained while children were still intubated. If they developed respiratory distress after extubation, the treating physician could decide to support them with HFNC according to our study protocol (Figure 1). This study was approved by the Seattle Children’s Hospital IRB and the Ethics Committee at Instituto Nacional de Salud del Niño.

## Preparation

### Identify Champions

Anyone who has attempted to enact change in an organization acknowledges the necessity of identifying local champions to oversee the intervention. Borrowing from the engineering industry, many health care organizations have adopted systems analysis and improvement approaches to improve the quality of health care delivery (9, 17). These principles focus on involving frontline health care workers in both identifying problems in the current system as well as suggesting possible solutions to streamline the process. Prior studies in resource-limited settings have shown that participation of local providers in the quality improvement process leads to more effective and sustainable solutions (18). Although we had access to a HFNC protocol from a resource-rich setting, we knew that direct implementation of that protocol would not be effective. Instead, we formed a core group of PICU providers at INSN to lead the HFNC project and used the protocol as a starting point to talk with locals to understand the process of caring for pediatric patients with respiratory failure at INSN.

### Map the Process

Process mapping is the systematic creation of flow maps to understand how patients move through the health care system and is used to identify bottlenecks and prioritize interventions. To be most effective, all stakeholders involved in the system should be represented in the final process map. We did not use formal process mapping during the preparation phase. Instead, a core group of INSN physicians and respiratory therapists with nearly 20 years experience in direct patient care in the PICU at INSN helped our team understand how HFNC could fit into their process of providing advanced respiratory care. This local perspective allowed us to design a HFNC protocol appropriate for their environment, increasing the chance of a successful intervention. Because implementation occurred in the context of a physician-led research study, nurses were not included in the early preparation phase. In retrospect, the addition of nurses to the core group from the beginning would have been extremely helpful in identifying potential issues for bedside providers prior to HFNC introduction.

### Determine Resource Needs

Prior to implementing any new technology for advanced respiratory care, it is essential to determine the availability of resources in the particular environment. As others have suggested, use of advanced respiratory care is only appropriate in settings that have the capability to closely monitor vital signs including oxygenation, have adequately trained staff, and have all equipment necessary to provide the specific type of respiratory support (19). Given that INSN has enough nurses to maintain a nurse:patient ratio of 1:2 and is able to provide invasive and non-invasive mechanical ventilation at each bed within the PICU, we focused on procurement of HFNC equipment and staff training. Detailed discussion about the selection of HFNC system is beyond the scope of this article, but gathering information about upfront and ongoing supply costs in addition to established processes for equipment purchasing and maintenance should inform discussions with local leadership. Ultimately, the choice of HFNC system should be left to local experts.

### Training

Local staff training is paramount to successful implementation, and the specific training module should be tailored to the needs of the specific environment. Adapting to the local context is part of the pre-implementation action cycle described by the Knowledge to Action Framework (20), which has guided implementation studies from Canada to the Democratic Republic of Congo (21). Because the educational needs of physicians and nurses are different, we chose to develop separate training modules for each group. The physician module provided more details regarding scientific evidence for HFNC mechanisms of action and efficacy in different patient populations, whereas the nursing module provided a summary of the evidence with more practical aspects of HFNC setup and management. We trained physicians first because they would be the ones to make the decision to use HFNC, and we knew their buy-in would be required in order to start using the technology. We then trained nurses over the course of 1 week, with morning and afternoon training sessions daily so that they could attend during scheduled shifts, as there is no mechanism in place to compensate them for additional hours spent on training. Although somewhat onerous, this adaptation was required in order to reach as many nurses as possible. All participants completed pre- and post-tests to both evaluate the quality of the training sessions as well as assess knowledge acquisition.

### Identify Potential Barriers

The next pre-implementation step in the Knowledge to Action Framework is to identify and address any barriers to knowledge use (20). We used qualitative methods to explore these barriers, conducting focus groups with nurses and one-on-one semistructured interviews with physicians after the training and before the introduction of HFNC. Qualitative research methods gather information in more depth, allowing participants to expand upon ideas, which are then organized into themes using thematic analysis (22). Some of the most prevalent themes were innate to the local health care system. For instance, the frequency of physician handoffs and a siloed health care system with insufficient interdisciplinary communication make it challenging to create a uniform, longitudinal care plan for patients. These issues have been reported in a variety of other health care contexts (23, 24), but having these up-front discussions with front-line providers helped us strategize our implementation plan to ensure success.

## Implementation and Sustainability

### Interrogate the Process

After the 18-month preparation period, we were ready to introduce HFNC for pediatric respiratory failure at INSN with the goal of shortening the duration of invasive mechanical ventilation. It is important to recognize that many interventions that are effective in research studies fail to translate into improved patient outcomes. To address that discrepancy, implementation research emphasizes the need to evaluate the effectiveness of the implementation process in addition to monitoring the primary outcome of the intervention. In real life scenarios, the implementation process has a direct effect on whether an intervention achieves its desired outcome. Determinant frameworks, such as the Consolidated Framework for Implementation Research, can be used to understand factors that influence the success of an intervention. This framework describes five important components: the intervention, the individuals involved, the inner and outer settings, and the implementation process (25). Given that these components will influence the success of deployment, it is important to develop ways to assess each of these factors throughout the implementation process. Each component is discussed in more detail below.

### The Intervention

**Figure 2:**
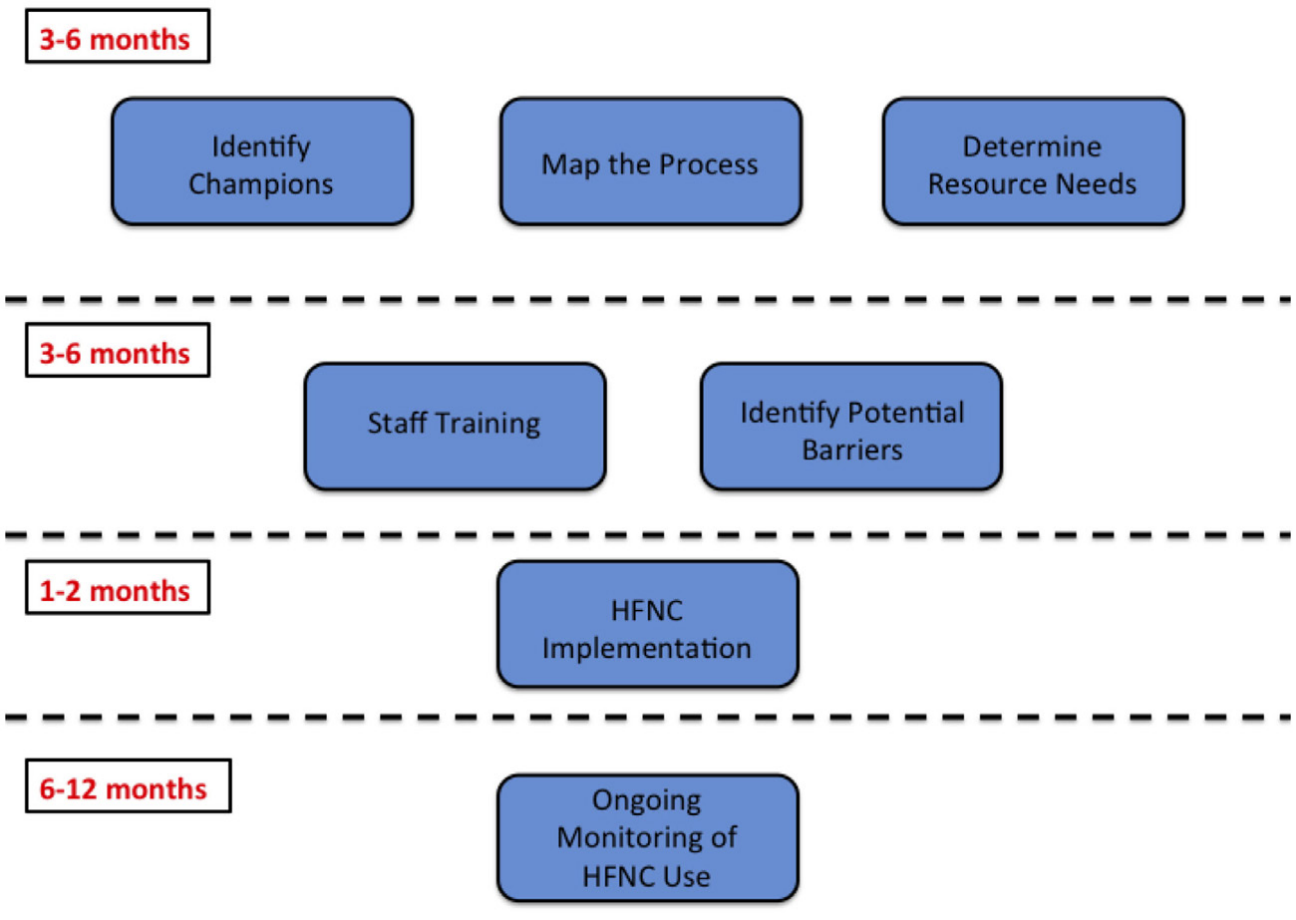
High flow nasal cannula implementation timeline.

As we learned from the focus groups, the intervention itself must be seen as beneficial for locals. Not all interventions that are effective in resource-rich settings are appropriate for resource-limited settings, and all implementation should be guided by local needs. In our case, the desire for HFNC came from INSN providers who had observed challenges with management of non-invasive ventilation, including skin breakdown, difficulty maintaining a seal around the mask, and relative scarcity of equipment. Even with this local buy-in, the planning phase lasted approximately 1 year to ensure the intervention would meet local needs (Figure 2). If considering implementation of an intervention in a new resource-limited setting, it is essential to spend adequate time up-front building a strong collaboration to maximize the chances of success. In addition, checking in with the local team periodically can identify any unanticipated challenges that need to be addressed. During our follow-up focus group discussions, we learned that it was difficult for staff to remember the details of the HFNC protocol. To address this, we hung large flow charts of the protocol throughout the PICU to serve as a resource for clinicians.

### The Individuals

The individuals involved in the intervention play a key role in the outcome of the intervention. Above, we emphasized the importance of local champions in the planning process, but these individuals need to demonstrate strong leadership throughout the intervention. Our core group of PICU providers filled many different rolls throughout the intervention. They responded to questions about HFNC, organized ongoing training sessions, encouraged local “hold-outs” to try using HFNC, and addressed any new issues that arose. In addition, the individuals using the intervention are also key players in its outcome. Adopting new practices is challenging for people in any setting, but in resource-limited settings, it can sometimes seem impossible. This can lead to resistance to change, which is why it is so important to have local leaders who can lead by example. Finally, a lack of familiarity with a new technology can contribute to hesitancy to use it. By organizing regular training sessions, nurses and physicians had the opportunity to ask questions related to their experience and go over specific case studies to highlight important aspects of HFNC management. After the training sessions, many nurses reported a desire for more frequent opportunities to review competencies, even beyond HFNC use. This emphasizes the importance of incorporating periodic review sessions to any implementation project to ensure individuals continue to feel comfortable with the new technology and/or protocol.

### The Inner and Outer Settings

The inner and outer settings also influence the effectiveness of a new technology. Although the differences can be subtle, the inner setting generally consists of the structural, political, and cultural context in which the implementation will take place. The outer setting encompasses the greater economic, political, and cultural context surrounding the organization responsible for implementation (26). In Peru, this meant determining whether or not the Ministry of Health would be involved in our project since INSN is under their control. The Ministry of Health had minimal influence over our study; however, in other settings, government oversight may play more of a role and these representatives need to be included in regular organizational meetings. The leadership structure and culture of the PICU at INSN includes both physician and nursing leaders who we engaged prior to implementation. One unanticipated challenge we faced was an unexpected PICU medical director leadership transition, and the position was filled with interim individuals for several months. Fortunately, these individuals were also supportive of our study; however, this transition could just as easily have been detrimental to the success of our project. This highlights the necessity of extra efforts during times of transition to ensure the inner and outer settings remain supportive.

### The Implementation Process

Finally, the implementation process itself greatly impacts the outcome of the intervention. This is where the work of the core group of providers at INSN paid off. With their recommendations, we implemented HFNC during respiratory season to increase the chance of finding eligible patients. Initially, the uptake of the intervention was slow, with some physicians hesitant to change their practice and adopt new technology. However, with on-site support of colleagues involved in the implementation process, HFNC use increased over time. Similar to other interventions, as providers witnessed successes with HFNC and its ease of use, they were more willing to try it for other patients. The type of support the implementation team provides must fit within the cultural context of the clinical setting. In some settings, direct hands-on guidance may be welcomed whereas in other settings, this may be perceived as interfering with clinical care. At INSN, the local team provided “behind-the-scenes” support, meaning they provided subtle suggestions about trying HFNC for some patients while emphasizing that the ultimate decision was up to the treating physician. Again, this emphasizes the importance of guidance by a team of local champions to determine what is appropriate for their setting.

### Sustainability

After implementation, it is important to maintain ongoing support for HFNC use. This includes many of the concepts described above: having a group of local experts to address clinical concerns, organizing ongoing staff training, and maintaining a consistent supply chain. At INSN, HFNC was implemented in the context of a research project with a specific research protocol. Over the 17-month study period, 29 patients received HFNC support for post-extubation respiratory failure. Study enrollment closed November 30, 2017, so our team of local experts is currently working on developing a protocol for HFNC use for general clinical care. This process will also be more successful if implementation science principles are utilized.

## Conclusion

Overall, our implementation experience at INSN has shown that HFNC can be successfully introduced in resource-limited settings. Utilizing tools of implementation science to engage key stakeholders during the planning process, understand the local process and identify its unique challenges, recruit local champions to facilitate training and support throughout the implementation process, and check-in with providers periodically after implementation greatly increases the chance of successful implementation. It is important to recognize that the timeline will likely be slower than anticipated, so maintaining momentum throughout the process is essential to keep local stakeholders engaged. As with all new interventions, sustainability is challenging and requires substantial ongoing effort to maintain. Our experience has taught us that with a core group of dedicated individuals, changing practice to improve the care of critically ill children is possible.

## Author Contributions

All authors contributed to the design of the work as well as the drafting and/or revising of this work and have approved this final version for publication.

## Conflict of Interest Statement

KN received travel expenses from Fisher & Paykel, manufacturer of high flow nasal cannula equipment, to attend an educational conference. All other authors declare that the research was conducted in the absence of any commercial or financial relationships that could be construed as a potential conflict of interest.
